# Mitophagy: A novel perspective for insighting into cancer and cancer treatment

**DOI:** 10.1111/cpr.13327

**Published:** 2022-10-05

**Authors:** Congkuan Song, Shize Pan, Jinjin Zhang, Ning Li, Qing Geng

**Affiliations:** ^1^ Department of Thoracic Surgery Renmin Hospital of Wuhan University Wuhan China; ^2^ Department of Emergency Taihe Hospital Shiyan China

## Abstract

**Background:**

Mitophagy refers to the selective self‐elimination of mitochondria under damaged or certain developmental conditions. As an important regulatory mechanism to remove damaged mitochondria and maintain the internal and external cellular balance, mitophagy plays pivotal roles in carcinogenesis and progression as well as treatment.

**Materials and methods:**

Here, we combined data from recent years to comprehensively describe the regulatory mechanisms of mitophagy and its multifaceted significance in cancer, and discusse the potential of targeted mitophagy as a cancer treatment strategy.

**Results:**

The molecular mechanisms regulating mitophagy are complex, diverse, and cross‐talk. Inducing or blocking mitophagy has the same or completely different effects in different cancer contexts. Mitophagy plays an indispensable role in regulating cancer metabolic reprogramming, cell stemness, and chemotherapy resistance for better adaptation to tumor microenvironment. In cancer cell biology, mitophagy is considered to be a double‐edged sword. And to fully understand the role of mitophagy in cancer development can provide new targets for cancer treatment in clinical practice.

**Conclusions:**

This review synthesizes a large body of data to comprehensively describe the molecular mechanisms of mitophagy and its multidimensional significance in cancer and cancer treatment, which will undoubtedly deepen the understanding of mitophagy.

## Graphical abstract

Mitophagy is a physiological process regulated by complex and diverse mechanisms. Here we summarize the 15 main molecular mechanisms involved in the mammalian mitophagy processes and some potential anticancer drugs targeting mitophagy.

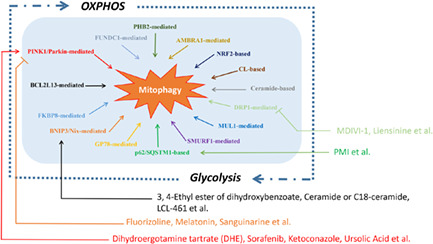

## 1 INTRODUCTION

Mitochondria are the main sites of cellular oxidative phosphorylation, and are considered to be the power source of cellular metabolism, and the ATP generated by mitochondria is the main source of energy for cellular life activities. During their evolution, mitochondria have acquired numerous functions, including energy metabolism, regulation of calcium homeostasis, phospholipid biosynthesis and transport, redox signalling and heme generation.^1^, ^2^, ^3^ When mitochondria are subjected to stress stimuli such as nutrient deficiency, hypoxia, DNA damage, inflammation and the use of mitochondrial uncouplers, damaged mitochondria can lead to the release of reactive oxygen species (ROS) or apoptotic factors, resulting in cellular damage or apoptosis.^4^, ^5^, ^6^, ^7^ In this case, mitochondria can be cleared in the form of selective autophagy, a process called mitophagy. Mitophagy and mitochondrial biogenesis are two opposing physiological processes that are particularly important in maintaining the normal function and quantity of mitochondria and cell stability.^8^ In order to meet the needs of mitochondrial turnover and to cope with a variety of stressful environments, mammalian cells have evolved multiple diverse molecular mechanisms to regulate the appropriate number and quality of mitochondria at different stages of translation and post‐translation.^9^, ^10^, ^11^

Mitophagy involves the process of selectively targeting mitochondria by autophagosomes for mitochondrial degradation.^12^ Several proteins responsible for mitophagy have been found to be involved, including Parkin (a protein encoded by PARK2 gene), BNIP3, BNIP3L/Nix, FUNDC1, etc. Mitophagy has been demonstrated to provide beneficial effects in eliminating damaged or redundant mitochondria to maintain mitochondrial network and cellular homeostasis. For example, it can remove paternal mitochondria during zygote formation, and mitochondria during erythrocyte maturation and muscle differentiation, etc.^13^, ^14^ An increasing number of studies have reported the close relationship between mitophagy and various diseases, including cancer.^15^, ^16^, ^17^ Cancer is a global puzzle, and the effects of mitophagy on cancer are multifaceted, and the specific role of mitophagy in different cancers, as well as in different cancer stages, remains largely unknown. Fully analysing the close relationship between mitophagy and cancer can provide new valuable reference for cancer treatment strategy. This review combined with data from recent years to comprehensively describe the regulatory mechanisms of mitophagy and its multifaceted significance in cancer, and addressed the potential of targeting mitophagy as a cancer therapeutic strategy.

## 2 MOLECULAR REGULATORY MECHANISMS OF MITOPHAGY

Mitophagy is a physiological process regulated by complex and diverse mechanisms. Currently, there are two major molecular regulatory mechanisms for mitophagy in mammals,^18^ the first is Parkin‐dependent, which involves PINK1/Parkin‐mediated mitophagy, and the second is Parkin‐independent with mitophagy mediated by BNIP3/Nix, FUNDC1 et al. With the deep investigation of mitophagy, other non‐classical pathway‐mediated mitophagy mechanisms have been gradually revealed, such as BCL2L13, PHB2, FKBP8 and DRP1‐mediated mitophagy. Further exploration of these regulatory mechanisms can help further understanding the mechanisms of disease and provide strategies for finding new therapeutic avenues.

### 2.1 PINK1/Parkin‐mediated mitophagy

**FIGURE 1:**
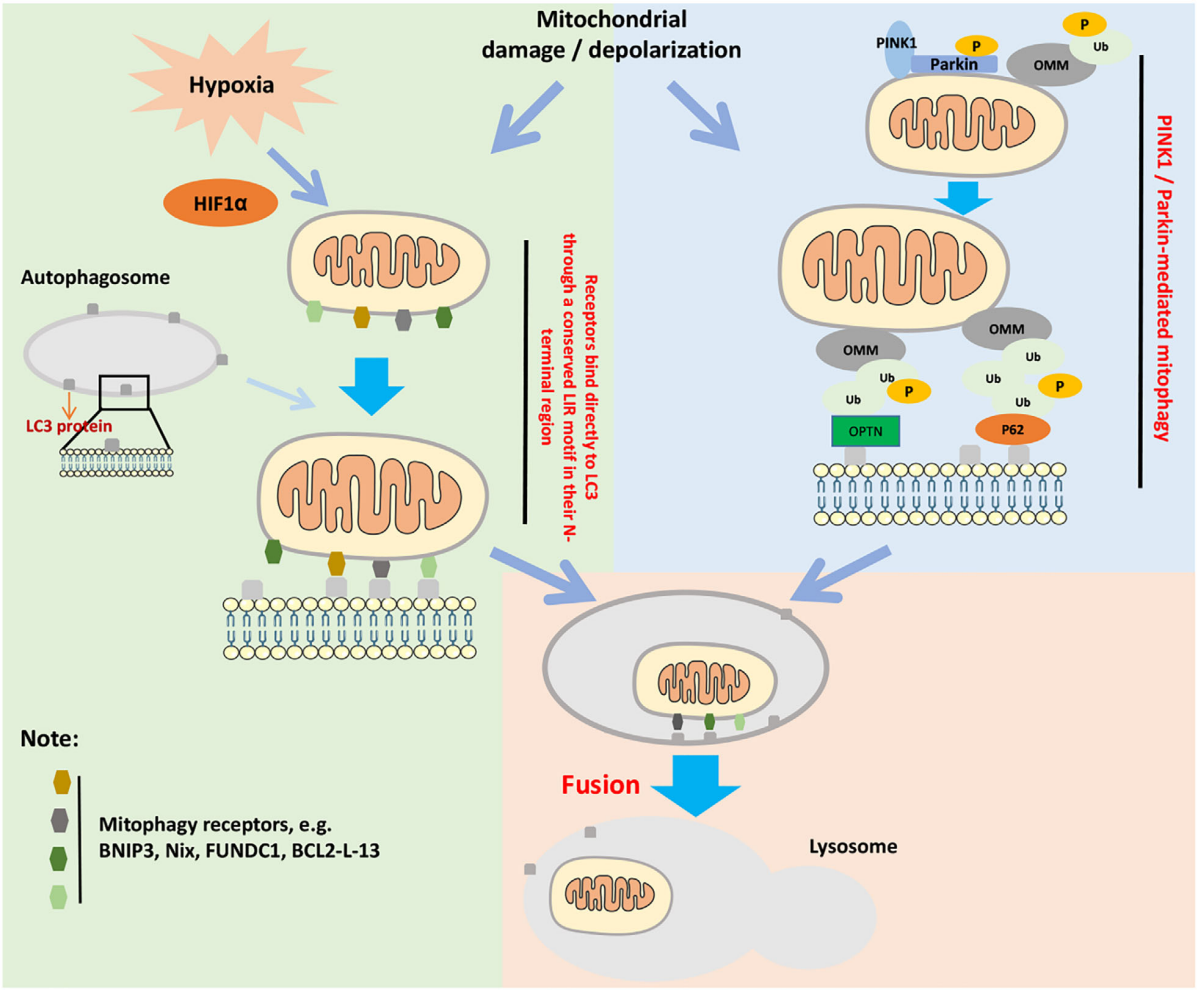
Major molecular mechanisms of mitophagy. The left side of the figure reflects receptor (mainly BINP3, Nix, FUNDC1, BCL‐2L‐13) mediated mitophagy. These mitochondrial receptors mediate mitophagy by binding directly to LC3 on autophagosomes through a conserved LIR motif in their N terminal region. Hypoxia is an important stimulus in inducing this process. The right side of the figure reflects PINK1/Parkin‐mediated mitophagy. This mitophagy process occurs in an ubiquitination‐dependent form, and ubiquitination of specific mitochondrial proteins enhances the ability of PINK1 to phosphorylate ubiquitin on mitochondrial proteins to recruit mitophagy receptors and mediate mitophagy. After further polyubiquitination, Parkin recruits adaptor proteins (such as p62/SQSTM1, OPTN) and interacts with LC3 on the surface of the autophagosome membrane to promote mitophagy.

The PINK1/Parkin pathway has been proposed as the main regulatory mechanism of mitophagy.^19^ PINK1 and Parkin play important roles in regulating mitochondrial functional integrity, and their mutation or impairment can lead to abnormal mitochondrial aggregation. The serine/threonine kinase PINK1 contains an N‐terminal mitochondrial targeting sequence that binds to mitochondria to induce mitophagy.^20^ Under normal conditions, PINK1 is translocated into the inner mitochondrial membrane (IMM) via the outer membrane translocase and inner membrane translocase complex (TOM/TIM complex), which is subsequently cleaved by the protease PARL on the IMM, and is further degradation by other proteases within the mitochondria.^21^ The mitochondrial membrane potential and intact structure affect PINK1's translocation to the IMM. Impaired mitochondria is bound to destroy the structure of the mitochondrial membrane or change the membrane potential, which inhibits the translocation of PINK1 to the IMM,^21^ and also suppresses the degradation of PINK1. PINK1 accumulates on the outer mitochondrial membrane (OMM) and phosphorylates ubiquitin on mitochondrial proteins to recruit mitophagy receptors such as p62, OPTN and NDP52. These receptors contain an LC3 interaction region (LIR) that interacts directly with LC3 on autophagosomes, for degrading damaged mitochondria through autophagolysosomes.^22^ Moreover, PINK1 can also recruit Parkin (the Parkin protein is located in the cytoplasm and is an E3 ubiquitin ligase) to phosphorylate at serine 65 of the ubiquitin‐like domain of PINK1,^23^ enhancing the activity of Parkin's E3 ubiquitin ligase, enabling mitochondrial proteins (such as VDAC1, MARF, MIF1 and MIF2) ubiquitination. The ubiquitination of these specific mitochondrial proteins in turn enhances the phosphorylation of ubiquitin on mitochondrial proteins by PINK1 to recruit mitophagy receptors and mediate mitophagy.^24^ Further polyubiquitination of Parkin then recruits the p62/SQSTM1 adaptor protein and interacts with LC3 on the phagocytic membrane surface to promote mitophagy^25^ (Figure 1).

The PINK1/Parkin pathway, as an important regulatory mechanism of mitophagy, is also affected by multiple molecules. Deubiquitinases such as USP8 have been shown to directly ubiquitinate Parkin itself to enhance its stability and induce mitophagy.^26^ Parkin substrates, however, can be directly deubiquitinated by USP15 and USP30, thereby removing ubiquitin moieties from mitochondrial proteins to prevent mitophagy.^27^, ^28^, ^29^ Furthermore, phosphatase and tensin homologue (PTEN)‐long (PTEN‐L) has been reported to dephosphorylate PINK1 and ubiquitinate mitochondrial proteins to inhibit the mitophagy triggered by the PINK1/Parkin pathway.^30^ Anti‐apoptotic proteins (e.g., Mcl‐1, Bcl2l1/Bcl‐xl, Bcl‐w) can prevent the mitochondrial ubiquitination activity of Parkin by disrupting or inhibiting the link between PINK1 and Parkin.^31^ Several pro‐apoptotic proteins, such as Puma, Bim and Noxa, cause Parkin to translocate to mitochondria, promoting mitophagy.^16^ Additionally, TANK‐binding kinase 1 (TBK1) stimulates the phosphorylation of autophagy receptors to form signal amplification loops to regulate the selective autophagy process of damaged mitochondria.^32^, ^33^ TBK1 can also directly phosphorylate Rab7A through the PINK1/Parkin pathway to promote mitophagy. Moreover, AMBRA1 is also involved in recruiting Parkin to mitochondria to induce p62/SQSTM1‐dependent mitophagy.^34^ Overall, PINK1/Parkin‐mediated mitophagy involves a complex program, and interfering with each of these steps may affect mitophagy.

### 2.2 BNIP3/Nix‐mediated mitophagy

Unlike Parkin/PINK1‐mediated mitophagy, BNIP3 and Nix (BNIP3L)‐mediated mitophagy are independent of mitochondrial ubiquitination. BNIP3 and Nix, as key mitochondrial adaptors, can directly interact with mitochondria and target autophagosomes. BNIP3 is a hypoxia‐inducible molecular adaptor.^35^, ^36^ Upon hypoxia stimulation, BNIP3 is upregulated and anchored to the OMM through its C‐terminal transmembrane domain, which is essential for its localization to mitochondria, while its N‐terminal is exposed to the cytosol.^37^ Normally, BNIP3 exists as an inactive monomer in cytosol, but upon stress, BNIP3 forms a stable homodimer through its C‐terminal transmembrane domain and anchors to the OMM.^38^, ^39^ Nix has been reported to show 53%–56% homology to BNIP3, and plays a key role in mitochondrial clearance during erythrocyte maturation.^40^ Due to the high sequence similarity between BNIP3 and Nix, expression of BNIP3 can also restore mitochondrial clearance in Nix‐deficient reticulocytes.^41^ Like other mitophagy receptors, a conserved LIR exists in the N‐terminal region of BNIP3 and Nix (Figure 1) that phosphorylates key residues on their serine/threonine (e.g., Ser34, Ser35 of Nix and Ser17, Ser24 of BNIP3) to interact with LC3‐II on the nascent isolation membrane.^42^, ^43^

The molecular mechanisms regulating BNIP3/Nix‐mediated mitophagy have not been fully elucidated. Hypoxia is considered to be a typical stress factor that induces BNIP3/Nix‐mediated mitophagy. Under hypoxia, HIF1, the most important transcription factor for maintaining oxygen homeostasis, is upregulated, activates the transcription and expression of BNIP3, and competitively dissociates the Bcl2/Beclin‐1 complex, enabling the release of Beclin‐1 to form a complex containing PI3K‐III. While through the PI3K/AKT pathway, this complex can regulate autophagy‐related proteins to activate mitophagy.^44^, ^45^ However, the hypothesis that hypoxia activates Nix has been challenged. In some studies, the transcription of BNIP3 and Nix was significantly up‐regulated under hypoxia, but Nix sensitivity to hypoxia was significantly reduced,^46^, ^47^ and Nix expression was neither associated with the oxygen partial pressure in tumours nor activated by ischemia in brain cells.^48^, ^49^, ^50^ Therefore, we speculate that in addition to hypoxia, there may be other key factors in activating Nix transcription, such as DNA methylation,^51^ miRNA regulation,^52^, ^53^ additional transcription factors (E2F‐1, FOXO3, TP53, etc.),^54^, ^55^, ^56^ NF‐KB, etc. Existing data support that Nix expression can be regulated at both pre‐ and post‐transcriptional levels^43^, ^48^, ^57^ and triggered by different cellular stress environments, including but not limited to hypoxia. Interestingly, from the structure of BNIP3 and Nix, the key residues on the serine/threonine in the N‐terminal region (such as Ser34, Ser35 of Nix and Ser17, Ser24 of BNIP3) are phosphorylated sites, and phosphorylation is the main molecular switch that controls these proteins to promote mitophagy,^58^ which provides diversity and selectivity for regulating BNIP3/Nix‐mediated mitophagy.

### 2.3 FUNDC1‐mediated mitophagy

As a mitophagy receptor protein, FUNDC1 also plays an important role in mediating mitophagy. FUNDC1 is a tertiary transmembrane protein that is localized to the OMM. Similar to BNIP3 and Nix, FUNDC1 directly binds to LC3 through a conserved LIR motif in its N‐terminal region to mediate mitophagy.^59^ FUNDC1‐mediated mitophagy is mainly regulated by phosphorylation and dephosphorylation at Ser13 and Tyr18 residues located in the LIR motif. In general, Ser13 and Tyr18 residues can be phosphorylated by CK2 and Src kinases accordingly, and FUNDC1 in the phosphorylated state interacts weakly with LC3. Under hypoxia, on the one hand, the protein phosphatase PGAM5 dephosphorylates Ser13 of FUNDC1,^60^ and on the other hand, as Src kinase and CK2 activities decrease, the level of FUNDC1 phosphorylation is reduced, promoting the binding of FUNDC1 to LC3 and mitophagy.^59^ Moreover, under hypoxia, ULK1 kinase, a key component of the autophagy initiation complex, moves to mitochondrial FUNDC1 and phosphorylates its Ser17 to enhance the interaction of FUNDC1 with LC3,^11^ thus promoting mitophagy.

The present data suggest that post‐translational modifications of FUNDC1, especially ubiquitination and phosphorylation, can well regulate FUNDC1‐mediated mitophagy. MARCH5 is the much‐studied E3 ubiquitin ligase that can regulate FUNDC1 expression. MARCH5 is an integral OMM protein with four transmembrane segments that are involved in the control of mitochondrial morphology. Overexpression of MARCH5 results in mitochondrial lengthening. MARCH5 binds to mitofusin 2 (MFN2) and Drp1, and is involved in mitochondrial fusion and cleavage, respectively.^61^ MARCH5 ubiquitinates FUNDC1 at Lys119 sites, and during hypoxia FUNDC1 expression is reduced in an ubiquitin‐proteasome‐dependent manner, whereas endogenous knockout of MARCH5 impairs FUNDC1 ubiquitination and degradation.^62^ p53 is a regulator of mitophagy and mitochondrial fission. It has been reported that p53 can upregulate CK2 expression and induce FUNDC1 phosphorylation inactivation at Ser13, thereby inhibiting mitophagy. In this process, DNA‐dependent protein kinase catalytic subunit (DNA‐PKcs) plays a key role, which activates and phosphorylates p53, thereby enhancing Drp1 transcription, and also inhibits the mitophagy required for FUNDC1.^63^ The above studies confirmed that NR4A1 is an upstream signal of DNA‐PKcs activation and p53 upregulation. Another study also observed that NR4A1 can induce phosphorylation inactivation of FUNDC1, thereby inhibiting mitophagy.^64^ In addition, there is also a potential link between Ripk3 and FUNDC1 mediated mitophagy that following microcirculation reperfusion, Ripk3 is upregulated, leading to FUNDC1 inactivation by post‐transcriptional modification of the FUNDC1 phosphorylation sites.^65^ BCL2L13 / Bcl‐xl, an anti‐apoptotic domain‐containing BH3 molecule, is also known to regulate FUNDC1 mediated mitophagy, which can bind to the mitochondrial phosphatase PGAM5 and inhibit the PGAM5‐FUNDC1 interaction to prevent the dephosphorylation of FUNDC1 at Ser13 to block FUNDC1‐mediated mitophagy.^66^ In addition, the targeted regulation of miR‐137 is also believed to regulate FUNDC1‐mediated mitophagy,^67^ and this regulation is mainly carried out by affecting the expression of mitophagy receptors (Nix and FUNDC1).^68^ FUNDC1, as a relatively new mitophagy receptor, its involvement in mediating mitophagy still needs to be further revealed.

Overall, mitophagy mechanisms are complex and diverse. In addition to PINK1/Parkin pathway, BNIP3/Nix pathway, FUNDC1 pathway, there are many other molecules or pathways (BCL2L13, PHB2, FKBP8, DRP1, etc.) that can also affect mitophagy. In Table 1, we summarized a number of relatively well‐studied mitophagy mechanisms. Globally, there is crosstalk between mitophagy mediated by different pathways. For example, BNIP3 interacts with PINK1 and promotes PINK1 accumulation on OMM, leading to Parkin translocation to mitochondria.^88^ Nix is ubiquitinated by Parkin, thus promoting the targeting of selective autophagy adaptor NBR1 and the formation of autophagosomes around mitochondria,^89^ which suggests that under different influence conditions, multiple pathways tend to mediate mitophagy in concert rather than in isolation.

**TABLE 1:** Molecular regulatory mechanisms of mitophagy

Mitophagy pathways	Properties and distribution of key moleculars	Mitophagy processes
PINK1/Parkin‐mediated mitophagy	PINK1 is a serine/threonine kinase, and Parkin is an E3 ubiquitin ligase. They are the key inducers of mitophagy.	The procedure as detailed can be seen in the main text section. It can be regulated by deubiquitinases (USP8, USP15, USP30)^26^, ^27^, ^28^, ^29^ and PTEN‐L.^30^
BNIP3/Nix‐mediated mitophagy	Both BNIP3 and NIX are hypoxia‐inducible molecular adaptors, and distributed on the outer mitochondrial membrane (OMM).	The procedure as detailed can be seen in the main text section. It can be regulated by HIF‐1a, DNA methylation, miRNAs, transcription factors (E2F‐1, FOXO3, TP53, etc.), NF‐KB.^54^, ^55^, ^56^
FUNDC1‐mediated mitophagy	FUNDC1, an OMM protein, acts as a receptor for mitophagy.	The procedure as detailed can be seen in the main text section. Post‐translational modifications of FUNDC1, especially by ubiquitination and phosphorylation, can well regulate FUNDC1‐mediated mitophagy.^62^, ^66^
BCL2L13‐mediated mitophagy	BCL2L13 is distributed in the OMM, and is a mammalian homologue of the yeast Atg32.	BCL2L13 binds directly to LC3 through its LIR motif, thereby inducing mitophagy. It can be regulated by phosphorylation (Ser272).^69^, ^70^
FKBP8‐mediated mitophagy	FKBP8 acts as a multifunctional adaptor with antiapoptotic activity, and is distributed on OMM.	The FKBP8 contains a LIR motif that directly interacts with LC3 to mediate mitophagy.^71^
PHB2‐mediated mitophagy	PHB2 is distributed in the inner mitochondrial membrane (IMM), and is an anti‐proliferative protein.^72^	PHB2 binds to LC3 and promotes autophagosomes to envelop damaged mitochondria.^73^ It can regulate PINK1/Parkin pathway by controlling PINK1 stability via the PARL‐PGAM5 axis, which is independent of the LC3‐binding capacity.^74^
AMBRA1‐mediated mitophagy	The AMBRA1 pool localizes to mitochondria. It can act as a mitophagy receptor.	AMBRA1 directly interacts with LC3 through its LIR motif to stimulate mitophagy, and it is also involved in the recruitment of Parkin to mitochondria to induce p62‐dependent mitophagy mediated by PINK1/Parkin.^34^ AMBRA1‐mediated mitophagy is still poorly studied, and lack consensus.
DRP1‐mediated mitophagy	Dynamin‐related protein 1 (DRP1) is a member of the protein kinesin superfamily.^75^ DRP1 participates in the division of mitochondria and is usually found in the cytoplasm in physiological conditions.^76^	Drp1 hydrolyzes GTP through its GTPase activity, which in turn destroys IMM and OMM, leading to mitochondrial fission.^77^ Drp1 can also interact with mitophagy receptors such as FUNDC1 and BCL2L13 to induce mitophagy.^78^
MUL1‐mediated mitophagy	The mitochondrial E3 ubiquitin ligase 1 (MUL1) is an E3 ubiquitin ligase anchored to the OMM. Its amino terminus with RING finger structure is located in cytoplasm.	MUL1 binds to GABA receptor‐associated protein (GABARAP) through its LIR motif, located in the RING finger domain of MUL1.^79^ However, the specific mechanism of MUL1‐mediated mitophagy has not yet been fully elucidated.
SMURF1‐mediated mitophagy	SMAD ubiquitination regulatory factor 1 (SMURF1) is a HECT‐domain ubiquitin ligase, and has a dual role in autophagy with its C2 domain.^80^	SMURF1 was rather linked to selective autophagy of intracellular bacteria, termed xenophagy. SMURF1‐mediated mitophagy is still poorly studied, and many mechanisms remain unknown.
GP78‐mediated mitophagy	Glycoprotein 78 (GP78) is located in the mitochondria associated endoplasmic reticulum (ER) domain, and is an ER reticulum anchored ubiquitin ligase (E3).^18^	GP78 induces mitophagy upon mitochondrial depolarization by recruiting LC3 to the GP78‐positive ER domain.^81^ GP78‐induced mitophagy is still poorly studied, and many mechanisms remain unknown.
p62/SQSTM1‐based mitophagy	The p62/SQSTM1 functions as an autophagy adapter.	The translocation of p62/SQSTM1 to mitochondria enhances multiubiquitination of OMM proteins, which in turn recruits LC3 to initiate mitophagy.^82^ p62/SQSTM1‐based mitophagy is still poorly studied, and many mechanisms remain unknown.
NRF2‐based mitophagy	NRF2 is considered to be a major regulator of cellular redox homeostasis, and can affect mitochondrial function.^83^	NRF2 can antagonize the Warburg effect to increase mitophagy stability.^84^ The specific mechanism of NRF2‐based mitophagy still needs further investigation.
CL‐based mitophagy	Cardiolipin (CL) is an intra‐mitochondrial phospholipid involved in the stabilization of the mitochondrial membrane and mitochondrial cristae, and is localized in the OMM.	CL directly interacts with LC3 when mitochondria are damaged to initiate mitophagy, independent of mitochondrial membrane depolarization.^85^, ^86^
Ceramide‐based mitophagy	Ceramides is a complete ER membrane protein consisting of a sphingosine backbone and a fatty acyl chain.	Ceramide induces LC3B‐II‐mediated autophagosome targeting of mitochondria to initiate mitophagy.^87^

## 3 RELATIONSHIP BETWEEN MITOPHAGY AND CANCER DEVELOPMENT

Since mitochondria play key roles in energy production, calcium homeostasis, phospholipid synthesis, redox signalling, heme production and apoptosis,^1^, ^2^, ^3^ mitochondrial dysregulation is closely related to a variety of human diseases. Mitophagy plays important roles in cancer cell homeostasis and tumour progression, acting as either pro‐cancer or anti‐cancer factor.^4^, ^5^ While the normal level of mitophagy is a defensive protective mechanism of the body to different environmental pressures, excessive abnormal mitophagy may cause abnormalities in the mitochondrial network and disorders of energy metabolism, which undoubtedly creates conditions for the occurrence and progression of cancer. Mitophagy plays a double‐edged role in cancer, and its specific role is associated with different cancer types as well as with different cancer stages.^90^ In the following sections, we will focus on the relationship between mitophagy and various cancer types to reveal the role of mitophagy in tumorigenesis and progression.

### 3.1 PINK1, Parkin, and cancer

PARK2 is a special gene encoding the Prakin protein, located on human chromosome 6q25.2–27 within FRA6E. This locus is prone to genomic alteration,^91^ which is also an important cause of Parkinson's disease in adolescents. PARK2 is recognized as a tumour suppressor, and is often absent in multiple cancers.^92^ A previous study^93^ reported that, up to 33% of colorectal cancer patients had loss of heterozygosity for PARK2, and a consistent phenomenon was observed in another study.^94^ Deletion of a single allele of PARK2 in Apc^Min/+^ mice accelerated the development of adenoma,^93^ implying that PARK2 mutations may drive tumour progression. This may be related to the fact that mutation in PARK2 gene abolishes Parkin's E3 ligase activity. In addition, a previous study^95^ also found that PARK2 knockout mice were highly susceptible to spontaneous hepatocellular carcinoma (HCC), in which PARK2‐deficient hepatocytes exhibited abnormal proliferation and resistance to apoptosis. Another study^93^ reported that PARK2 overexpression could inhibit the proliferation of colon cancer cells, and the E3 ligase activity of Parkin may be a key factor in its tumour suppressive effect. Additionally, the occurrence and development of breast cancer was also associated with defects in mitophagy. Deletion of the PARK2‐containing FRA6E fragile region was common in breast cancers, and Parkin expression was significantly down‐regulated in a variety of breast cancer cell lines as well as in primary breast cancer tissues. Restoring Parkin expression in Parkin deficient cell lines significantly reduced their tumour biological behaviour,^96^ suggesting that PARK2 deletion was a specific advantage in causing tumour growth, which further supported the suppressive effect of Parkin on tumours. Moreover, PARK2 deletion was also common in lung cancer. Mutations in PARK2 resulted in the inhibition of mitophagy and the upregulation of ROS and cyclin E, which were thought to be tumour drivers in lung cancer.^97^ However, in different cancer contexts, PARK2 deletion may have different or even completely opposite cancer effects. For example, in melanoma, Parkin deficiency can induce apoptosis and inhibit melanoma development and metastasis by inhibiting Mfn2 ubiquitination.^98^

PINK1 is considered to be a sensor of mitochondrial dysfunction, and plays important and extensive roles in tumorigenesis and development. In glioblasts, PINK1 can negatively regulate the growth of tumour cells, and the loss of its expression can stabilize HIF1a and then induce the production of ROS and tumour growth.^99^ However, PINK1 is upregulated in lung cancer^100^ and oesophageal cancer^101^ compared with normal tissues, and can promote the proliferation and chemotherapy resistance of tumour cells. Furthermore, it has been reported that iron transporters, such as SLC25A37 and SLC25A28, may be degraded by PINK1/Parkin‐mediated mitophagy, thus leading to the accumulation of iron in mitochondria,^102^ which can trigger a HIF1a‐dependent Warburg effect and inflammasome activation in tumour cells. In this context, the function loss of PINK1/Parkin accelerates the occurrence of pancreatic tumours driven by K‐ras mutant, suggesting that cancer occurrence and progression may be regulated by mitochondrial iron homeostasis.

### 3.2 BNIP3, Nix and cancer

BNIP3 and Nix are two important proteins mediating mitophagy and play key roles in tumorigenesis and development. BNIP3 is a pro‐apoptotic protein that can enhance mitophagy by inhibiting damaged mitochondrial fusion and making it more likely to be eliminated.^103^ Hypoxia has been considered as a key driver of cancer occurrence and development. Under hypoxia, HIF1a is activated, which in turn increases the expression of BNIP3. BNIP3 overexpression was frequently observed in human malignancies, such as salivary adenoid cystic carcinoma,^104^ endometrial carcinoma^105^ and breast ductal carcinoma in situ,^106^ and was associated with aggressive tumour biology and poor prognosis. In contrast, high expression of BNIP3 was associated with favourable outcomes in invasive breast cancer^106^ and laryngeal carcinoma.^107^ Moreover, the high expression of BNIP3 and Nix in precancerous lesions could serve as markers of progression in invasive cancers.^106^ However, it is worth noting that BNIP3 is not highly expressed in all tumours. For example, in colon cancer,^108^ gastric cancer,^109^ pancreatic cancer^110^, ^111^ BNIP3 often appears downregulated, which is thought to be associated with abnormal methylation mediated by DNA‐methyltransferase 3β (DNMT3β) and DNA‐methyltransferase 1 (DNMT1). BNIP3 downregulation accelerated the growth of colon cancer cells and weakened its sensitivity to chemotherapy.^108^ The same phenomenon was also seen in pancreatic cancer cells,^110^ which might be related to the cellular localization of BNIP3. For example, in glioblastoma cells,^112^ although the BNIP3 expression level was increased in the hypoxic region of the tumour, it was not localized in the mitochondria or cytoplasm, but in the nucleus, and its nuclear localization was a sign of tumour dormancy. Similarly, BNIP3 expression was also detected in the nucleus in non‐small cell lung cancer,^113^ cervical cancer,^114^ breast cancer^106^ and prostate cancer.^115^ Nuclear BNIP3 was also significantly correlated with a poor prognosis in non‐small cell lung cancer.^113^ Nuclear BNIP3 in invasive breast cancer was associated with smaller tumour size, lower tumour grade, and ER positivity, but not with survival. However, in breast ductal carcinoma in situ, nuclear BNIP3 was associated with a 3‐fold increased risk of recurrence and shorter disease‐free survival.^106^ The mechanism by which BNIP3 maps to the nucleus is currently unknown. Understandably, increased ROS results in increased expression of HIF1a and its target genes as well as increased glycolysis, thus promoting the Warburg effect and subsequent cancer progression. BNIP3 has been reported to be absence of expression in advanced pancreatic cancer,^110^ and the recovery of BNIP3 expression made pancreatic cancer cells tend to die.^111^ We reasonably speculate that BNIP3 activation may prevent malignant cell transformation by controlling ROS levels and maintaining HIF1a stability. This also reaffirms the key role of BNIP3 as a tumour suppressor. However, globally, it was not difficult to find that BNIP3 expression was dysregulated in many cancers, and whether BNIP3 acts as a tumour suppressor or a tumour promoter depends on the cancer context.

Currently, the contribution of Nix to cancer cells remains controversial. The available data indicate that under hypoxia, Nix competitively binds to BCL2 through its BH3 domain, destroys the BCL2‐BECN1 complex, and the released BECN1 promotes the formation of the autophagosome.^22^, ^44^ Nix was thought to be an oncogene that disrupted the interaction of BCL2 with BECN1, and Nix‐associated autophagy might be associated to survival of solid tumours in hypoxic environments, such as Nix promoting survival of glioblastoma and pancreatic cancer cells.^116^, ^117^ Nix can also mediate the translocation of Parkin proteins on mitochondria, thus playing an important role in PINK1/Parkin‐mediated mitophagy. Further investigation, the link between Nix‐mediated mitophagy and cancer cell survival has been gradually revealed. It must be emphasized that Nix‐associated mitophagy plays a dual role in cancer biology. It has been reported that Nix recruited TR3 to the mitochondria to cause melanoma cell death.^118^ In Ewing sarcoma, the endogenous degradation of Nix allowed the malignant cells to survive.^119^ These findings suggested that Nix could act as a tumour suppressor in certain tumours, and that its mediated mitophagy could affect cancer cell survival. Overall, Nix‐mediated mitophagy plays a complex role in the fate of cancer cells. Nix may play different roles in different cancer types as well as at different stages of cancer progression.

### 3.3 FUNDC1 and cancer

As another hypoxia‐induced mitophagy receptor, FUNDC1 also plays critical roles in regulating the occurrence and progression of cancer.^62^ In the cancer cells of early‐stage cervical cancer patients, FUNDC1 had higher expression levels than the normal cells, and high FUNDC1 expression was negatively correlated with the patient prognosis. While inhibition of FUNDC1 resulted in increased sensitivity of cancer cells to the ionizing radiation and cisplatin.^120^ In another study, mitophagy promoted by FUNDC1 upregulation provided a benefit for the survival of laryngeal cancer cells.^121^ In versely, in hepatocytes, the knockdown of FUNDC1 caused the accumulation of damaged mitochondria, the release of mitochondrial DNA, the activation of caspase‐1 and the excessive production of IL‐1B, which stimulated the occurrence and progression of HCC.^122^, ^123^ Globally, the effect of FUNDC1 on cancer cells depends on the cancer type. The positive correlation between FUNDC1 expression and tumour progression can be attributed to hypoxia, but different degrees of mitophagy in different cancer contexts as well as the complex cancer microenvironment should also be responsible for the diverse effects. Appropriate mitophagy can be considered as a protective process against apoptosis, but excessive mitophagy can also lead to non‐apoptotic cell death, and mitochondrial dysfunction caused by defects of mitophagy can also promote tumour growth.^124^, ^125^ It has been reported that mitophagy can improve cancer cell survival by regulating apoptosis or improving the overall function of mitochondria,^90^, ^116^, ^126^ and FUNDC1 can also increase vascular endothelial growth factor (VEGF) receptor production and improve the nutrient supply of tumour cells. For example, in cervical cancer,^120^ breast cancer^127^ and HCC,^128^ FUNDC1 could promote cancer progression and have detrimental effects on prognosis. In contrast, FUNDC1 could also resist the occurrence and progression of some cancers (HCC,^122^ ovarian cancer, lung cancer^128^), which might be related to the close connection between FUNDC1 and metabolic reprogramming and cellular plasticity in cancer.

## 4 RELATIONSHIP BETWEEN MITOPHAGY AND METABOLIC REPROGRAMMING, CELL PLASTICITY AND DRUG RESISTANCE IN CANCER

**FIGURE 2:**
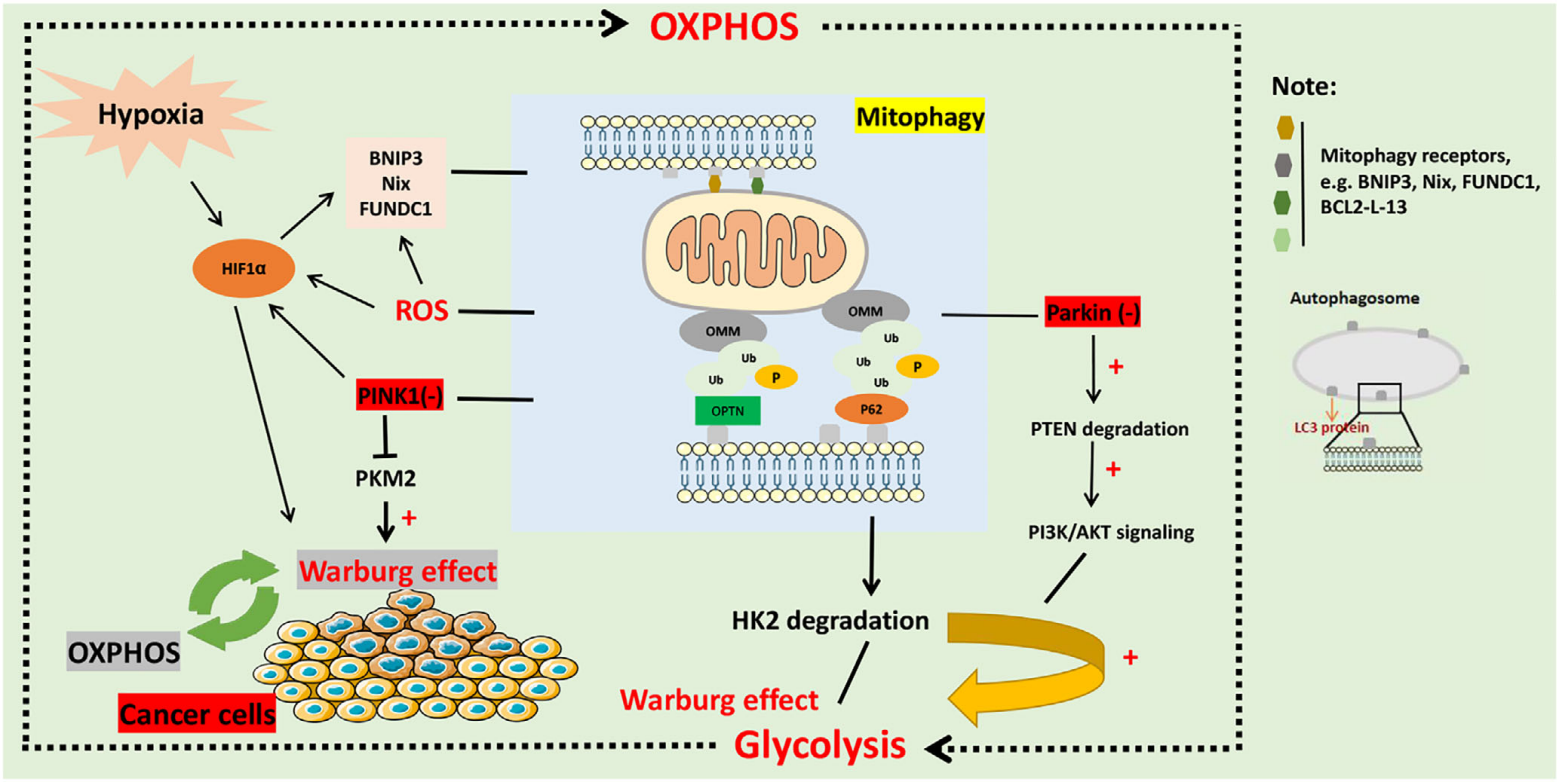
Mitophagy and metabolic reprogramming in cancer. Dysfunction of mitophagy is an important mechanism of energy metabolism reprogramming in cancer. Even with sufficient oxygen, cancer cells develop aerobic glycolysis to metabolize glucose to lactate, known as the Warburg effect. Parkin deletion promotes the degradation of PTEN, which in turn triggers PI3K/AKT signalling in cancer cells, and this canonical oncogenic pathway also accelerates aerobic glycolysis in cancer cells. Likewise, PINK deficiency or depletion can lead to Warburg effect by stabilizing HIF1a and reducing PKM2 activity to maintain rapid cancer cell proliferation. In addition, p62/SQSTM1‐dependent mitophagy can selectively degrade hexokinase 2 (HK2) to regulate glycolytic levels. Hypoxic stimulation, elevation and activation of mitochondrial ROS, induce transcriptional upregulation of HIF1a (which triggers glycolytic metabolism) and increased expression of its target genes (BNIP3, Nix, FUNDC1), which in turn regulates mitophagy.

Tumour microenvironment is the survival environment of tumour, which is composed of tumour cells, non‐tumour cells and extracellular matrix. In the process of tumorigenesis and progression, mitochondria are often overloaded to meet the rapidly growing demand for energy supply in order to cope with the changing and complex environmental pressure. Enhanced mitophagy in tumour cells may be an adaptive response to promote tumour growth. Mitophagy dysfunction is an important mechanism of cancer energy metabolism reprogramming.^129^ To maintain uncontrolled growth rates, cancer cells employ unconventional mechanisms to gain energy from the outside. Even with sufficient oxygen, cancer cells develop aerobic glycolysis to metabolize glucose to lactate, known as the Warburg effect.^130^ The energy acquisition of most cancer cells depends on glycolysis. Even in the presence of a large amount of oxygen, mitochondria will produce a considerable amount of ATP and reduce the use of oxidative phosphorylation (OXPHOS) mechanism. The expression and activity of key enzymes during glycolysis of cancer cells can obviously affect this metabolic process.^131^, ^132^ It was reported for p62/SQSTM1‐dependent mitophagy to selectively degrade hexokinase 2 (HK2) to control glycolytic levels. In HCC, autophagy deficiency leads to the upregulation of HK2 expression.^133^ In addition, autophagy‐related proteins can also affect the glycolysis process, thereby inhibiting the occurrence and progression of cancer. For example, the deletion of AGT5 activated the process of acute myeloblastic leukaemia (AML) development.^134^ Interestingly, the classical PINK1‐Parkin pathway and mitophagy mediated by BNIP3/Nix and FUNDC1 in response to hypoxia are also involved in glycolytic metabolism in cancer cells. For example, Parkin interacted with PTEN to regulate glycolysis, and Parkin deficiency promoted the degradation of PTEN, resulting in triggering PI3K/AKT signalling (the classical cancer‐related pathway) in cancer cells^94^ (Figure 2). In addition, degradation of SLC25A37 and SLC25A28 mediated by PINK1‐Parkin pathway can also lead to HIF1a‐dependent Warburg effect via increasing mitochondrial iron accumulation^102^ to maintain the rapid proliferation of cancer cells. It is clear that cancer occurrence is frequently associated with the activation of oncogenic signals as well as the hypoxia environment. Parkin has been reported to interact with the pyruvate kinase M2 (PKM2) to inhibit glycolysis,^135^ while it can also ubiquitinate and degrade HIF1a, thereby preventing its transcriptional targets, including glycolysis‐related proteins, from becoming activated.^135^ Similarly, PINK1 depletion leads to Warburg effects by stabilizing HIF1a and decreasing PKM2 activity^99^ (Figure 2). The interruption of key pathways or molecules mediating mitophagy, is bound to affect the normal play of mitophagy, and the increase and activation of mitochondrial ROS will induce transcriptional upregulation of HIF1a (which can trigger glycolysis metabolism) and its target genes (BNIP3, Nix, FUNDC1), thus regulating mitophagy^136^ (Figure 2). Although the current data support the close relationship between mitophagy and glycolytic metabolism of cancer cells, the reason for the increase of glycolysis of cancer cells is not completely clear. Mitochondrial dysfunction caused by mitophagy deficiency may be associated with Warburg effect, which is confirmed by the increase of aerobic glycolysis and accelerated tumour progression caused by the interruption of key pathways or molecules mediating mitophagy (such as PINK1 and BNIP3 deficiency).^137^ It has been shown that the tumours expressing activated KRAS relied on mitophagy to maintain sufficient metabolism under nutrient‐deficient conditions. And the insufficient energy supply caused by the loss of mitochondrial function caused by Kras‐induced mitophagy would be compensated for from the glycolytic pathway.^138^ Notably, the main advantage of the Warburg effect is not in the production of glycolytic ATP, but rather in the production of many glycolytic intermediates acting as precursors of anabolic processes, such as NADPH, ribose‐6‐phosphate, amino acids, lipids, etc. These substances can provide nutrients to cancer cells and meet their metabolic needs.^139^ In addition, mitosis may also play vital roles in the metabolic reprogramming of cancer cells. The continuous division and growth of cancer cells requires more energy. Mitosis promotes the glycolysis pathway, which is conducive to OXPHOS to meet the rapidly growing energy demand.^16^

Stem cells are a class of multipotential cells with self‐replication capacity. Cancer stem cells (CSCs) are a subset of cancer cells with stem cell characteristics. It has been reported that mitophagy played an important role in maintaining stem cell pools, including the stem cell properties of CSCs.^140^ For example, the maintenance of self‐renewal ability of haematopoietic stem cells benefits from PINK1/Parkin‐mediated mitophagy.^141^ Loss of PINK1/Parkin suppresses mitophagy in haematopoietic stem cells and reduces their stemness.^142^ Furthermore, the stemness of liver CSCs can also be maintained by mitophagy‐mediated selective degradation of p53.^143^ These findings suggest that mitophagy is closely related to cell plasticity. Interestingly, the switch between mitophagy‐mediated glycolysis and OXPHOS promotes the stem cell properties of CSCs.^144^ CSCs exhibit unique metabolic adaptation by regulating mitochondrial activities determined by the cellular environment. It has been reported that CSCs in nasopharyngeal carcinoma cells undergone metabolic reprogramming through mitochondria, and the conversion from OXPHOS to glycolytic metabolic pathway promoted and maintained the stemness of CSCs.^145^ Mitophagy‐mediated metabolic reprogramming may promote tumour progression while meeting energy supply. Drp1 is an important dynamin‐related GTPase for maintaining mitochondrial morphology. In brain tumours, Drp1 could be catalysed by cyclin‐dependent kinase 5 (CDK5) to maintain self‐renewal and tumour growth by initiating cell mitochondrial fragmentation.^146^ Damaired mitochondria are cleared from the CSC via the mitophagy pathway to maintain CSC stemness. Mitophagy may also retain the drug resistance of CSC while promoting and maintaining the CSC stemness and tumour growth. It was reported that higher levels of mitophagy can be observed in the CSC of colorectal cancer resistant to doxorubicin.^147^ Similarly, in the study of Mishra et al.,^148^ researchers also found that mitophagy promoted the stemness and chemical resistance of CSC in oral squamous cell carcinoma. Moreover, it has also been shown that downregulation of Drp1 activity by COX‐2 can inhibit stem cells and subsequently increase the sensitivity of nasopharyngeal darcinoma cells to 5‐fluorouracil.^149^ Overall, there was close relationship between mitophagy and CSC stemness and drug resistance. The mechanism and molecular link of mitophagy in regulating metabolic reprogramming, cellular plasticity and drug resistance in cancer cells still need further investigation.

## 5 MITOPHAGY AS A POTENTIAL TARGET FOR CANCER THERAPY

The above review clearly illustrates the key role of mitophagy in tumour genesis and development as well as in promoting and maintaining metabolic reprogramming, cell stemness and chemical resistance of cancer cells. On the one hand, proper mitophagy can eliminate damaged mitochondria, maintain cell homeostasis and normal metabolism, and promote cell survival to better adapt to the invasive environment.^124^ On the other hand, excessive mitophagy destroys normal mitochondria and impairs mitochondrial function, thus destroying cellular energy requirements and causing cell death.^125^ Targeting mitophagy pathway may affect the balance between tumorigenesis and cell death. Therefore, inhibition or induction of mitophagy is also a strategy worth considering in cancer treatment. In Table 2, we detailed the drugs that affected the role of mitophagy in various tumours.

**TABLE 2:** Drugs that affect mitophagy in various cancers

Drugs	Origin/characteristics	Mechanism of action	Cancer types
*Mitophagy inducers*			
Ceramide or C18‐ceramide	A central molecule of sphingolipid metabolism	BNIP3‐mediated mitophagy; directly interacts with LC3‐II to recruit autolysosomes to damaged mitochondria	HNSCC^150^; Glioblastoma^151^
LCL‐461	Ceramide analog drugs	The same as above	AML^152^
Dihydroergotamine tartrate (DHE)	Ergot alkaloid derivative	PINK1/Parkin‐mediated mitophagy; induction of apoptosis	Lung cancer^153^
Sorafenib	A multi‐kinase inhibitor	PINK1/Parkin‐mediated mitophagy; induction of apoptosis; ETC complexes II and III inhibition	Renal cancer; liver cancer^154^
Ketoconazole	An anti‐fungal drug	Downregulation of COX‐2 induces PINK1 accumulation; PINK1/Parkin‐mediated mitophagy; induction of apoptosis	HCC^155^
AT101	A novel BH3 mimic	Mitophagy; non‐apoptotic cell death	Glioblastoma^156^
Rapamycin	Anti‐fungal and immunosuppressants	Specific inhibitors of the mTOR protein; mitophagy	Renal cancer; breast cancer^157^
Curcumin	A yellow acidic phenolic substance derived from turmeric roots	Mitophagy	Nasopharyngeal carcinoma^158^
Valinomycin	Activable ion carrier	Damage the mitochondrial membrane potential by stimulating potassium flux; PINK1/Parkin pathway	HCC^159^, ^160^
PMI	A compound associated with p62‐mediated mitophagy	p62‐mediated mitophagy, ROS accumulation	HCC; neuroblastoma^82^
3,4‐Ethyl ester of dihydroxybenzoate	Errotinib intermediate; ethyl procatechate;	BNIP3‐mediated mitophagy	Oesophageal cancer^161^
Ursolic acid	A pentacyclic triterpenoid compound	PINK1/Parkin‐mediated mitophagy; reducing the mitochondrial membrane potential	Lung cancer^162^
Salinomycin	A potassium ionophore antibiotic that selectively inhibits the growth of Gram‐positive bacteria	Mitochondrial depolarization	Prostrate cancer^163^
Phenanthroline	A metal chelating agent that prevents streptozotocin induced chromosomal aberrations	Mitochondrial fragmentation and mitochondrial dysfunctions in a Drp1 dependent manner	Cervical carcinoma^164^
Ginsenoside	The active components of traditional Chinese herbal medicine Panax ginseng	PINK1/Parkin‐mediated mitophagy	Colon cancer^165^
Cannabidiol (CBD)	A nonpsychoactive ingredient from the cannabis plant	Mitochondrial dysfunctions; PINK1/Parkin‐mediated mitophagy	Chronic myeloid leukaemia^166^
*Mitophagy inhibitors*			
MDIVI‐1	An inhibitor of mitochondrial division	Drp1‐mediated mitophagy	Liver cancer^167^; Oophoroma^168^
Melatonin	Hormone, an endogenous indindlamine and antioxidant	PINK1/Parkin‐mediated mitophagy; downregulation of JNK kinase and Parkin, apoptosis induction	Cervical carcinoma^169^
PKI‐402	A potent inhibitor of PI3K and mTOR	Mitophagy	HCC^170^
Liensinine	An isoquinoline alkaloid	Drp1‐mediated mitophagy, blocks autophagosome‐lysosome fusion, apoptosis induction	Breast cancer^171^, ^172^
Sanguinarine	An isoquinoline alkaloid	PINK1/Parkin‐mediated mitophagy, disinterfere with the formation of late autolysosomes	HCC^173^
Fluorizoline	A synthetic molecule that can selectively target prohibitins	PINK1/Parkin‐mediated mitophagy	Cervical carcinoma; lung cancer^174^

The key role of mitophagy in inhibiting tumorigenesis and progression was detailed in the previous sections. Thus, the induction of mitophagy may be an effective therapeutic strategy to promote cancer cell death. Ceramide is a central molecule of sphingolipid metabolism, and is involved in regulating mitophagy. Activation of the ceramide stress pathway can induce mitophagy to promote cell death in HNSCC.^150^ In malignant gliomas, ceramide can upregulate BNIP3 expression to induce mitophagy and cause cancer cell death.^151^ A mitochondrial‐targeting ceramide analog (LCL‐461) was reported to effectively attenuate Kranolani (a FLT3‐ITD inhibitor) resistance by inducing lethal mitophagy in FLT3‐ITD^+^ AML in vitro and in vivo.^152^ Furthermore, overexpression of C18 ceramide or CerS1, also promoted aspartactase‐independent cell death and mitophagy.^175^ Dihydroergotamide tartrate (DHE), a horn alkaloid derivative, has been found to cause mitochondrial dysfunction, leading to activation of the PINK1/Parkin‐mediated mitophagy pathway, and subsequently lung cancer cell death.^153^ Similarly, in renal and liver cancer cells, sorafenib (a multikinase inhibitor) enhanced the occurrence of apoptosis via the mitophagy mediated by PINK1/Parkin pathway.^154^ As sorafenib was reported to stabilize PINK1 on the OMM to target and inhibit the electron transport chain in mitochondria, and activate the recruitment of Parkin by mitochondria to start mitophagy to treat liver cancer.^154^ In addition, it has been reported that ketoconazole (an antifungal drug) can induce apoptosis in HCC by downregulating COX‐2 to induce PINK1 accumulation as well as initiating PINK1/Parkin‐mediated mitophagy.^155^ And ketoconazole was also known to enhance the sensitivity of HCC cells to sorafenib and to cooperate with it to inhibit HCC growth.^155^ Further evidence of targeted mitophagy as a potential therapeutic strategy for HCC was consolidated in another study, which found that the loss of Yap (yes related protein) enhanced phosphorylation of JNK, triggering BNIP3‐mediated mitophagy.^176^ A novel BH3 mimic, AT101, has been reported to induce excessive mitophagy with non‐apoptotic cell death in glioma cells.^156^ Rapamycin had active therapeutic effects in kidney and breast cancers by promoting mitophagy.^157^ Curcumin was also shown to induce mitophagy in nasopharyngeal carcinoma cells induced by low‐intensity ultrasound therapy.^158^ Overall, this information suggests that lethal mitophagy is a key mechanism for tumour suppressor, and mitophagy inducers and their potential to treat cancer should therefore be the subject of future research.

As mentioned above, mitophagy also plays critical roles in promoting cancer cell survival and drug resistance, and thus inhibiting mitophagy is also an effective strategy for treating cancer. In most cases, targeting mitophagy not only inhibits the growth of cancer cells, but also enhances the role of anti‐cancer drugs. For example, melatonin, as an endogenous indoleamine and antioxidant, can inhibit mitophagy by downregulating Parkin, thus leading to apoptosis of cervical cancer cells. And melatonin can also inhibit the resistance of cancer cells to cisplatin, thus restoring the efficacy of chemotherapy.^169^ In addition, in HCC, MDIVI‐1 (mitochondrial division inhibitor 1) can impede Drp1‐mediated mitophagy and in turn enhance the efficacy of chemotherapeutic drugs.^167^ Meanwhile, the combination of MDIVI‐1 and TRAIL also enhanced TRAIL‐induced apoptosis in human ovarian cancer cells.^168^ Another study showed that in combination with dororubicin, MDIVI‐1 reduced the potential for cardiotoxicity,^177^ and the combination with MDIVI‐1 could also effectively overcome the resistance to cisplatin in tumour cells.^178^ These findings suggest that the combination of mitophagy inhibitors with certain anticancer drugs can significantly improve the effectiveness of chemotherapy. The following examples will further consolidate this conclusion. For example, the combination of the mitophagy inhibitor PKI‐402 with cisplatin clearly enhanced the sensitivity of HCC cells to cisplatin.^170^ Another known novel inhibitor of mitophagy, liensinin, enhanced the sensitivity of breast tumour cells to classical chemotherapeutic drugs (e.g., dororubicin, paclitaxel, vincristine and cisplatin) in vitro and in vivo.^171^, ^172^ Liensinine is an isoquinoline alkaloid, which induces the accumulation of mitophagosomes by inhibiting autophagosome lysosome fusion, and then activates mitochondrial division through Drp1/Dnm1,^172^ which may be a potential mechanism for liensinine to sensitize tumour cells to apoptosis induced by chemotherapeutic drugs. Notably, there is crosstalk between mitophagy and mitochondrial dynamics, which is diverse and complex. The upstream molecular mechanisms of mitochondrial fission and mitophagy are shared. A study from Zhou et al. has shown that bispectral protein phosphatase 1 (DUSP1) can inhibit mitochondrial fission required by Mff and BNIP3‐associated mitophagy through the JNK pathway, thereby alleviating cardiac ischemia–reperfusion injury.^179^ And the mitophagy mediated by FUNDC1 can also prevent the execution of mitochondrial fission and maintain mitochondrial homeostasis, ultimately conferring a renoprotective effect.^180^ These findings also imply that targeting mitochondrial homeostasis (fission/mitophagy) is a valuable potential strategy for treating certain diseases. Mitochondrial fission is mediated by dynamin‐related GTPase (Drp1) and mitochondrial fission factors (such as Mff). Whether mitochondrial fission is a necessary condition for the initiation of mitophagy remains controversial.^181^ However, it is undeniable that mitochondrial fission can promote mitophagy. For example, mitochondrial fragmentation caused by phenanthroline (an anticancer drug) promotes mitophagy.^164^ And MDIVI‐1 inhibits mitophagy by interfering with the process of mitochondrial breakage, which helps to improve the resistance of tumour cells to cisplatin.^178^ Theoretically, different stages of the mitophagy process can be targeted by genetic or pharmacological pathways to inhibit mitophagy, subsequently inhibiting tumour growth and enhancing anticancer sensitivity. For example, silencing mitophagy‐related genes (e.g., Parkin, PINK1, BNIP3, Nix, and FUNDC1), blocking the formation of autophagosomes and inhibiting the fusion of autophagosomes with lysosomes or the degradation ability of autophagosomes (e.g., liensinine) by PI3K inhibitors (e.g., 3‐methyladenine and LY294002).^172^, ^182^ It have shown that PINK1‐mediated mitophagy provided cancer cells with resistance to chemotherapy and that inhibition of PINK1 by RNA interference could make cells more sensitive to conventional chemotherapy.^101^ Similarly, inhibition of FUNDC1 also improved the sensitivity of cervical cancer cells to cisplatin.^120^ Globally, it was not difficult to find the importance of mitophagy in cancer drug resistance. Overall, mitophagy shows great potential as a very important target for cancer treatment. Inducing or inhibiting mitophagy can provide significant therapeutic benefits in cancer.

## 6 SUMMARY AND PROSPECT

Mitophagy is an extremely important cellular event, which is required for the regulation of cellular environmental homeostasis. As an important regulatory mechanism for cells to remove damaged mitochondria and maintain internal and external balance, mitophagy has significant impacts in the occurrence, progression and treatment of tumours. In cancer biology, mitophagy plays a double‐edged sword role, and its specific role varies with cancer types and stages. In this review, we summarize the important and cutting‐edge knowledge on the molecular processes that regulate mitophagy and how they affect cancer. However, several key issues remain to be addressed. For example, what is the specific mechanism and premise of different mitophagy pathways synergistically affecting the fate of cancer cells in the same cancer context? How does mitophagy drive the metabolic reprogramming of cancer cells, maintain the stemness of CSCs and promote chemoresistance, and is the specific mechanism affected by the cancer context? From the currently available data, in the search for new anti‐cancer drugs, targeting mitophagy may be an attractive option. Combining mitophagy inhibitors with chemotherapy may be a better approach for treating some cancers. However, in different cancer contexts and in the process of anti‐cancer drug treatment, how mitophagy is regulated has not yet been fully clarified. Whether activating or inducing mitophagy is a better strategy for cancer treatment still needs to be further investigated.

## AUTHOR CONTRIBUTIONS

Congkuan Song designed the study. Congkuan Song, Shize Pan and Jinjin Zhang consulted a large number of literature. Jinjin Zhang designed and drawn the figures and tables in the manuscript. Congkuan Song drafted the manuscript. Ning Li and Qing Geng reviewed and modified this manuscript. All authors read and approved the final manuscript.

## FUNDING INFORMATION

This work was supported by grants from the National Natural Science Foundation of China (No. 81770095, 81700093, 8210082163), the Fundamental Research Funds for the Central Universities (No. 2042021kf0081) and Science Fund for Creative Research Groups of the Natural Science Foundation of Hubei Province (No. 2020CFA027).

## CONFLICT OF INTEREST

The authors declare that they have no competing interests.

## INFORMED CONSENT

All authors consent to publication.

## Data Availability

Data sharing is not applicable to this article as no new data were created or analyzed in this study.
